# Plasma Cell Gingivitis With Cheilitis in an Adolescent: A Case Report

**DOI:** 10.7759/cureus.113604

**Published:** 2026-07-29

**Authors:** Aishwarya S Karnik, Kiran Kumar, Amey G Patil

**Affiliations:** 1 Department of General Dentistry, SDM College of Dental Sciences and Hospital, Dharwad, IND; 2 Department of Oral and Maxillofacial Pathology, SDM College of Dental Sciences and Hospital, Dharwad, IND; 3 Department of Restorative Dentistry, Rutgers School of Dental Medicine, Newark, USA; 4 Department of Diagnostic Sciences, Rutgers School of Dental Medicine, Newark, USA; 5 Center for Temporomandibular Disorders and Orofacial Pain, Rutgers School of Dental Medicine, Newark, USA

**Keywords:** cheilitis, diagnosis of exclusion, gingival enlargement, gingivectomy, histopathology (hp), hypersensitivity reaction, oral mucosal lesion, oral pathology, plasma cell gingivitis, plasma cell infiltrate

## Abstract

Plasma cell gingivitis (PCG) is an uncommon plasma-cell-rich inflammatory disorder of the gingiva that may clinically resemble plaque-associated enlargement, granulomatous disease, hematologic disease, or plasma-cell neoplasia. Its association with cheilitis is rare, and diagnosis requires clinicopathologic correlation.

An 18-year-old female presented with a 1-year history of progressive gingival enlargement and upper-lip swelling. Intraoral examination showed generalized erythematous marginal and papillary gingival enlargement with loss of stippling, bleeding on probing, pseudopocketing, and firm consistency. Extraoral examination showed diffuse, soft, non-tender swelling of the upper lip measuring approximately 1 × 3 cm. The initial clinical differential diagnosis included orofacial granulomatosis and puberty-associated gingival enlargement. Hematological screening showed anemia but did not show findings suggestive of leukemia or systemic infection. Incisional biopsy showed parakeratinized stratified squamous epithelium with pseudoepitheliomatous hyperplasia and a plasma-cell-rich inflammatory infiltrate composed of cytologically mature plasma cells without cytological atypia. Immunohistochemistry for kappa and lambda light-chain expression was not performed.

The patient had an inadequate response to non-surgical periodontal therapy and topical corticosteroids and was subsequently managed with gingivectomy. No disease-directed adjuvant therapy was administered after gingivectomy. At the one-month postoperative follow-up, both the gingival enlargement and upper-lip swelling had regressed. This case highlights the importance of biopsy and hematological screening in persistent gingival enlargement with associated cheilitis, particularly when clinical findings overlap with systemic or neoplastic mimics.

## Introduction

Plasma cell gingivitis (PCG) is an infrequent, benign inflammatory disorder of the gingiva defined histologically by a dense, predominantly subepithelial infiltrate of plasma cells within the lamina propria [1,2]. It belongs to a family of plasma-cell-rich mucosal reactions that have been described using several terms, including atypical gingivostomatitis, idiopathic gingivostomatitis, allergic gingivostomatitis, and plasmacytosis of the gingiva [1,3]. The condition preferentially involves the attached gingiva and can be diagnostically challenging because its clinical appearance overlaps with several inflammatory, granulomatous, hematological, and neoplastic conditions.

The prevailing view is that PCG represents a hypersensitivity reaction to an exogenous antigen. Historically, a cluster of cases described in the late 1960s and early 1970s was linked to a flavoring constituent of chewing gum, and discontinuation of the habit led to resolution [4]. Since then, a range of putative allergens has been implicated, including components of dentifrices, cinnamon and other spices, mint, red pepper, herbal tooth powders, and khat [5-7]. In a substantial proportion of patients, however, no causative agent is identified, and the lesion is regarded as idiopathic [2,8]. The contemporary understanding is therefore best framed as a non-specific, antigen-driven inflammatory response in a susceptible host rather than as a single disease with a uniform cause.

Clinically, PCG presents as diffuse erythema and edematous gingival enlargement, frequently with loss of stippling and a sharp demarcation at the mucogingival junction. Bleeding on minor provocation is common, and patients may report soreness aggravated by hot or spicy food or toothpaste [2,9]. Because the attached gingiva is involved, the differential diagnosis is broad and includes plaque-associated inflammatory enlargement; hormonally influenced enlargement, such as that seen in puberty and pregnancy; drug-induced enlargement; desquamative gingivitis; granulomatous conditions, including orofacial granulomatosis; and hematological or neoplastic disease [2]. Leukemic gingival infiltration, plasma cell mucositis, and plasma cell neoplasms, such as multiple myeloma, may clinically resemble benign PCG, making clinicopathologic correlation essential [2,10].

Histopathology is central to diagnosis. The benign nature of PCG is supported by cytologically mature plasma cells without architectural effacement and, where available, by immunohistochemical evidence of polyclonal kappa and lambda light-chain expression that helps exclude a monoclonal neoplastic infiltrate [2]. Reactive epithelial changes, including pseudoepitheliomatous hyperplasia, may accompany the infiltrate and should not be over-interpreted [11]. In practice, diagnosis requires correlation of clinical findings, hematological screening, and biopsy findings, particularly when systemic or neoplastic mimics are part of the differential diagnosis.

The association of PCG with cheilitis is rare. Kerr and colleagues originally described a triad of gingivitis, cheilitis, and glossitis [1], and Silverman and Lozada later characterized a comparable gingivitis-cheilitis-glossitis syndrome [8]; however, most subsequent cases of PCG have been reported in isolation. Documented examples of concurrent gingival and labial involvement remain few [9,12,13], and the mechanistic relationship between the two sites is not firmly established. This case is documented because PCG with cheilitis is an uncommon presentation that can mimic granulomatous, hematological, and plasma-cell neoplastic disease. It highlights the need for systematic exclusion of serious mimics, biopsy-based clinicopathologic correlation, and cautious interpretation when the causative allergen is not identified and ancillary immunohistochemistry is unavailable.

## Case presentation

An 18-year-old female presented in January 2020 with a 1-year history of swelling of the upper lip and gingiva. The swelling was reported to have appeared abruptly and to have increased gradually thereafter, reaching its presenting size. Her medical history was within normal limits, with no relevant comorbidities and no medication history associated with gingival enlargement. Directed history regarding commonly reported triggers for plasma cell gingivitis, including chewing gum, flavored dentifrices, cinnamon- or spice-containing products, mouthrinses, herbal tooth powders, and khat use, did not identify a consistent exogenous trigger. Formal allergy testing or patch testing was not performed.

**Figure 1:**
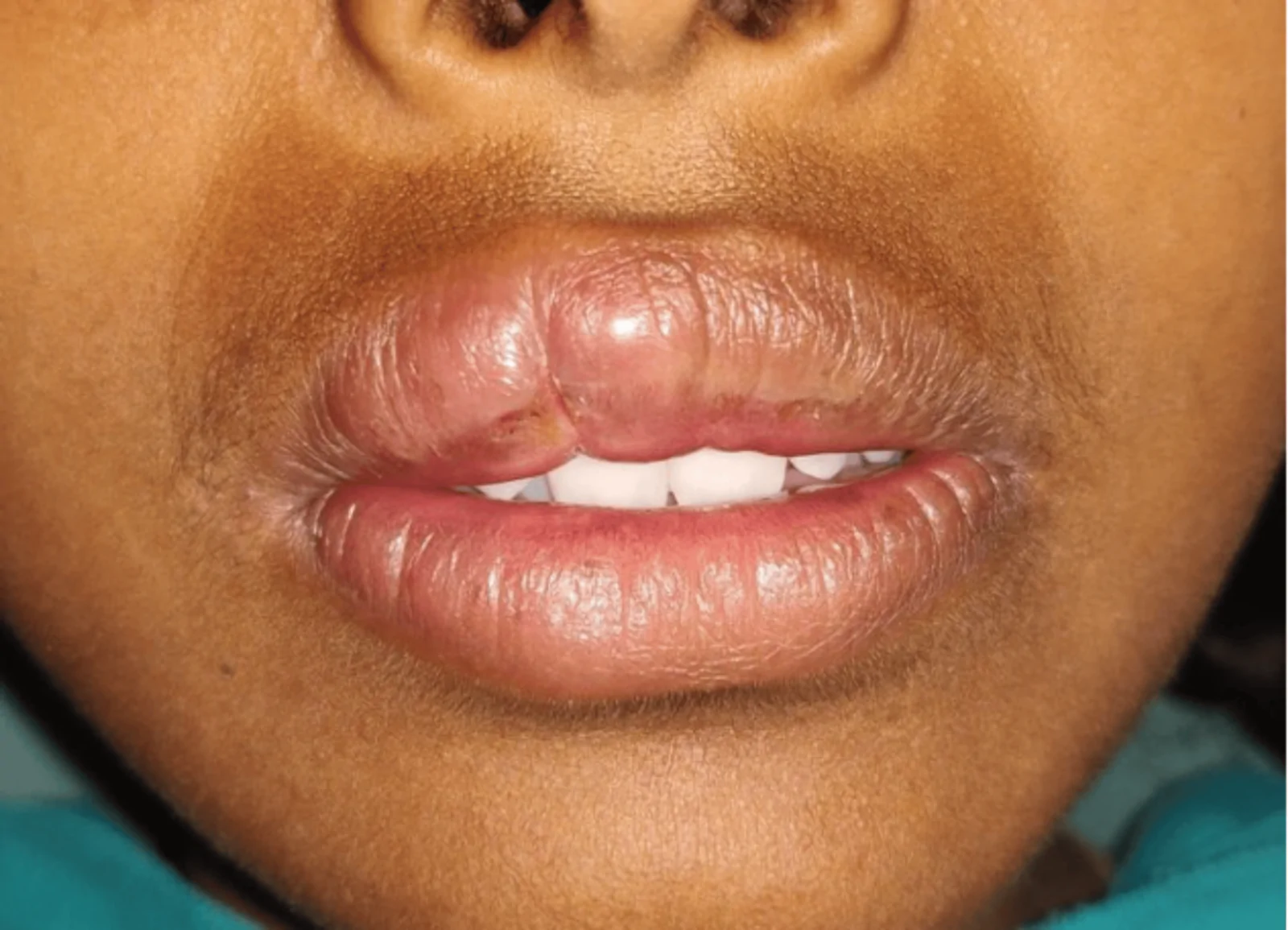
Extraoral photograph at presentation showing diffuse swelling of the upper lip (cheilitis).

Extraoral examination revealed diffuse swelling of the upper lip extending from the midline to the right commissure and, on the left side, to approximately 4 mm from the midline, measuring approximately 1 × 3 cm. The overlying skin appeared normal, with no discharge. On palpation, the swelling was soft and non-tender (Figure 1).

**Figure 2:**
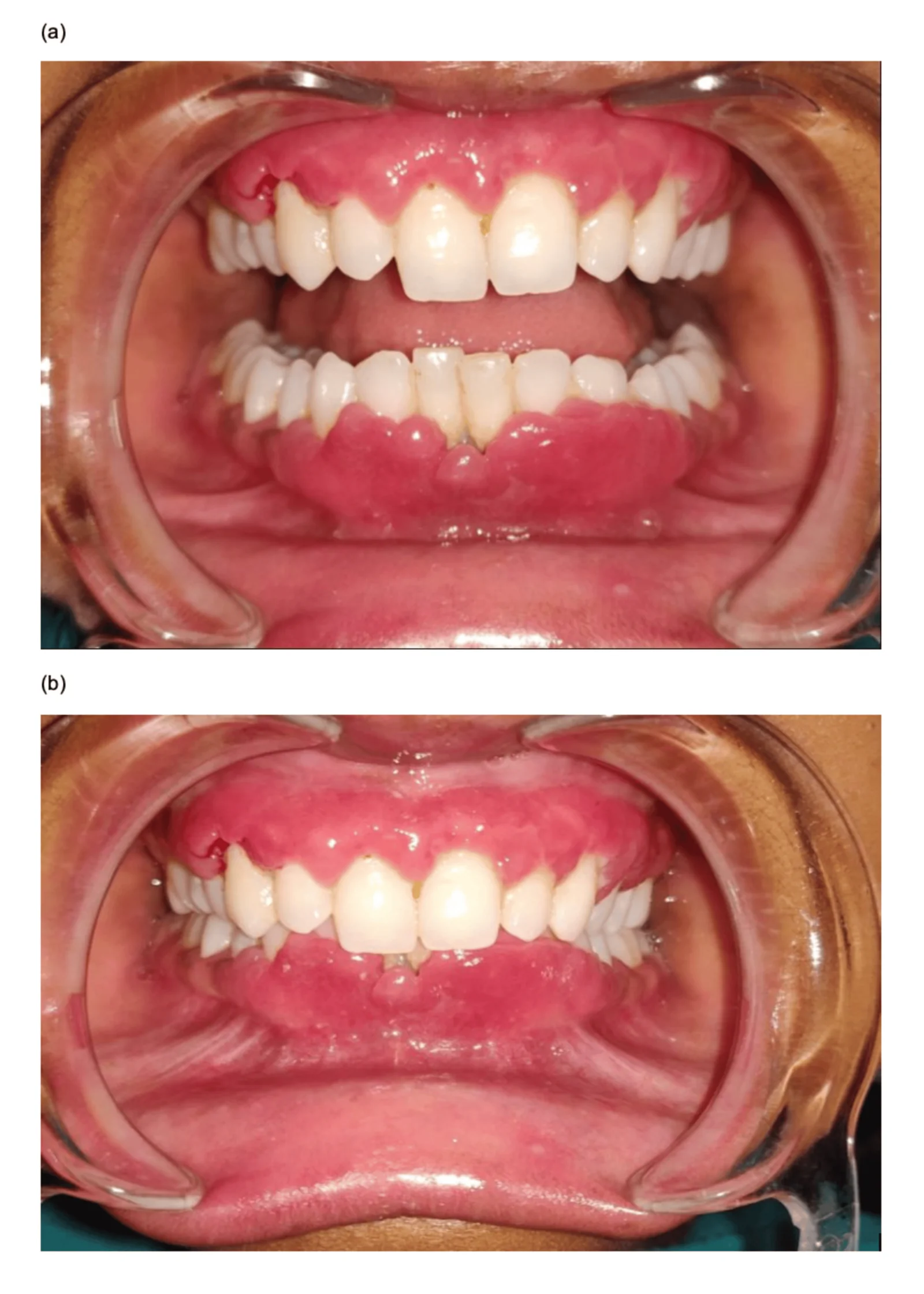
Intraoral retracted views demonstrating generalized marginal and papillary gingival enlargement with diffuse erythema, a multilobular surface, and loss of stippling (a) View in slight opening; (b) view in occlusion Areas of spontaneous bleeding are visible in the maxillary anterior region.

Intraoral examination revealed generalized gingival enlargement involving the marginal and papillary gingiva of both the maxillary and mandibular arches. The gingiva was bright red and multilobular, with loss of surface stippling. Bleeding on probing was present, and pseudopockets were noted. On palpation, the enlargement was firm and non-tender (Figure 2).

Based on the patient’s age, generalized distribution of enlargement, and concurrent labial swelling, the provisional differential diagnosis included orofacial granulomatosis and puberty-associated, hormonally influenced gingival enlargement.

Hematological investigation was undertaken to screen for systemic and hematological disease. Hemoglobin was 7.6 g/dL, consistent with anemia; bleeding time was 1 minute, and clotting time was 4 minutes 45 seconds, both within accepted limits. The total leukocyte count was 5,800 cells/mm³, and the differential count showed neutrophils 56%, lymphocytes 36%, eosinophils 5%, and monocytes 3%. The peripheral smear showed a dimorphic picture, consistent with anemia and most compatible with a nutritional cause. Random blood glucose was 81 mg/dL, and serology for human immunodeficiency virus (HIV)-1, HIV-2, and hepatitis B surface antigen (HBsAg) was negative. The normal leukocyte counts and unremarkable peripheral smear provided no evidence of a leukemic process, and the anemia was regarded as an incidental finding. The laboratory investigations and corresponding biological reference values are summarized in Table 1.

**Table 1:** Laboratory investigations and biological reference values HIV: human immunodeficiency virus; HBsAg: hepatitis B surface antigen Reference ranges may vary slightly among laboratories; the values shown are approximate commonly accepted biological reference intervals.

Investigation	Patient value/result	Biological reference value	Interpretation
Hemoglobin	7.6 g/dL	Female: approximately 12.0–15.0 g/dL	Low; consistent with anemia
Bleeding time	1 minute	Approximately 1–6 minutes	Within normal limits
Clotting time	4 minutes 45 seconds	Approximately 2–8 minutes	Within normal limits
Total leukocyte count	5,800 cells/mm³	Approximately 4,000–11,000 cells/mm³	Within normal limits
Neutrophils	56%	Approximately 40–70%	Within normal limits
Lymphocytes	36%	Approximately 20–40%	Within normal limits
Eosinophils	5%	Approximately 1–6%	Within normal limits
Monocytes	3%	Approximately 2–8%	Within normal limits
Peripheral smear	Dimorphic picture	No immature/blast cells expected	Compatible with anemia; no leukemic picture reported
Random blood glucose	81 mg/dL	Approximately 70–140 mg/dL	Within normal limits
Human immunodeficiency virus 1 and 2 serology	Negative	Negative	Non-reactive
Hepatitis B surface antigen	Negative	Negative	Non-reactive

No radiological investigation was performed before biopsy. After clinical examination and hematological screening, incisional biopsy was performed to establish a tissue diagnosis because the lesion was persistent, clinically non-specific, and associated with labial swelling.

**Figure 3:**
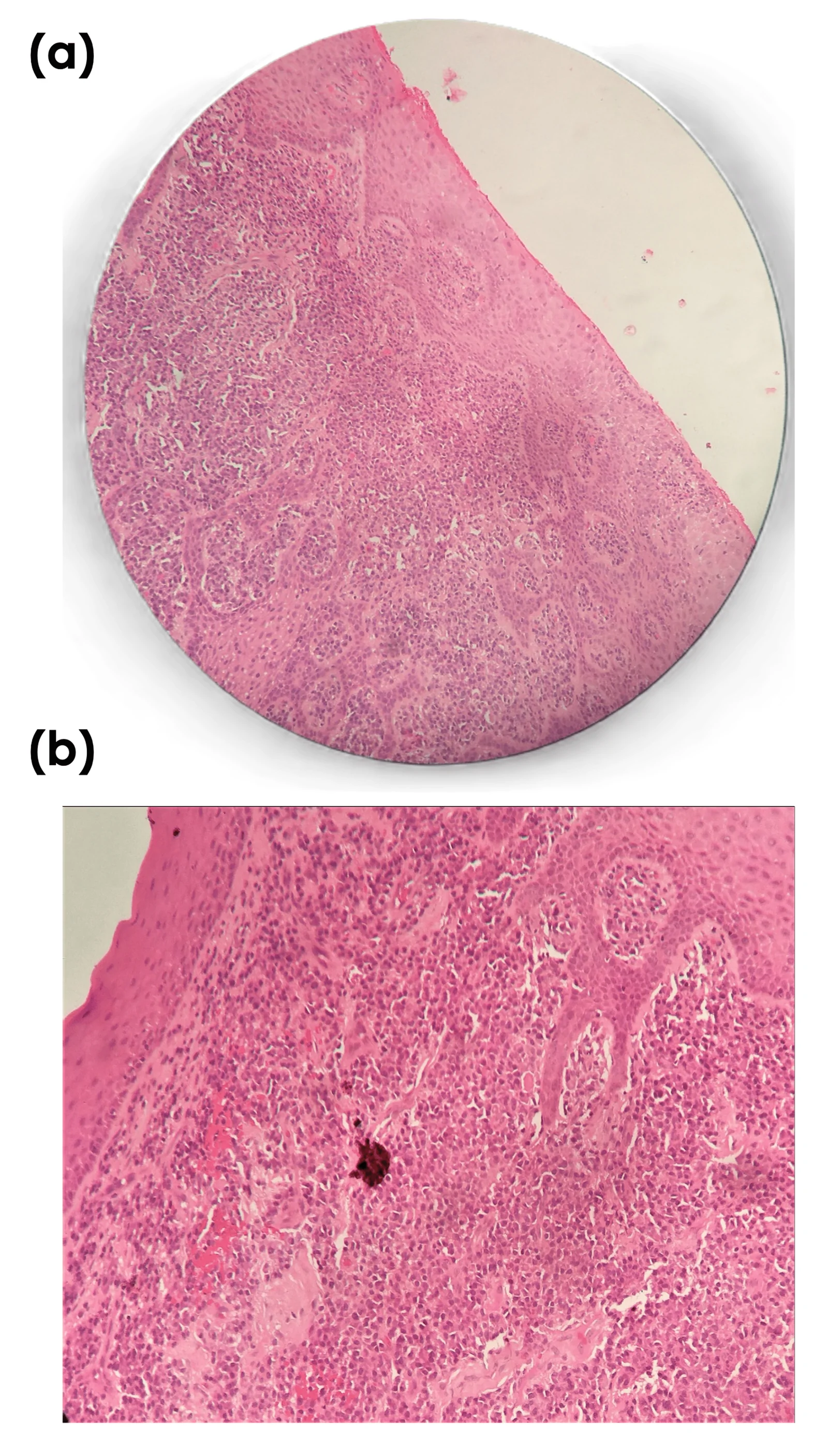
Available photomicrographs of the incisional gingival biopsy stained with hematoxylin and eosin (H&E) (a) Low-power view showing parakeratinized stratified squamous epithelium with reactive epithelial change and a dense underlying mononuclear/plasma-cell-rich inflammatory infiltrate expanding the lamina propria. (b) Higher-power view showing pseudoepitheliomatous epithelial hyperplasia with a dense plasma-cell-rich inflammatory infiltrate in the fibrocellular connective tissue. The reproduced photomicrographs are limited in resolution; therefore, the diagnosis was based on the original histopathologic interpretation and clinicopathologic correlation rather than the reproduced images alone.

The biopsy specimen showed parakeratinized stratified squamous epithelium ranging from atrophic to hyperplastic, with areas of pseudoepitheliomatous hyperplasia. The underlying fibrocellular connective tissue contained collagen bundles, fibroblasts, vascular stromal spaces, and a dense plasma-cell-rich chronic inflammatory infiltrate that was prominent beneath the epithelium and extended into the underlying stroma. The plasma cells were described as cytologically mature, with eccentric nuclei, cartwheel chromatin, and abundant cytoplasm, without cellular atypia (Figure 3).

Immunohistochemistry (IHC) for kappa and lambda light-chain expression was not performed; therefore, polyclonality could not be confirmed immunohistochemically. However, the absence of cytological atypia, unremarkable leukocyte counts and peripheral smear, and postoperative clinical regression supported a benign reactive plasma-cell process rather than a neoplastic plasma-cell disorder.

Correlating the clinical presentation, hematological screening, and plasma-cell-rich biopsy findings without cytological atypia, the findings supported a clinicopathologic diagnosis of plasma cell gingivitis associated with cheilitis. As a causative allergen could not be identified and formal patch testing was not performed, the lesion was classified as idiopathic on the available evidence.

Management comprised supragingival and subgingival scaling followed by a trial of topical corticosteroids. Because the lesion did not respond adequately to corticosteroid therapy, gingivectomy was undertaken. At the one-month postoperative follow-up in February 2020, regression of both the gingival enlargement and upper-lip swelling was observed on clinical examination. No disease-directed adjuvant therapy was administered after gingivectomy. No postoperative complications were documented during the available follow-up period. The patient was advised to continue structured follow-up to reinforce oral hygiene, reassess possible allergen exposure, and monitor for recurrence.

## Discussion

Disease overview

Plasma cell gingivitis is an uncommon benign mucosal condition characterized by a plasma-cell-rich infiltrate of the gingival lamina propria, and reported experience consists largely of single case reports and small series [2,10,11]. Although the disorder can affect a wide age range, the present patient, an adolescent, falls within the younger end of the reported spectrum [9,14], and the involvement of the attached gingiva is consistent with the disease’s recognized tropism [2]. The pathogenesis is best understood as an exaggerated immune response to an antigenic stimulus in a predisposed individual, producing the plasma-cell-rich infiltrate that defines the entity.

Immunologic and hypersensitivity mechanisms

The dominant etiological hypothesis is that plasma cell gingivitis represents an antigen-driven hypersensitivity reaction of the gingival mucosa. Reported triggers include chewing gum flavoring agents, dentifrice components, cinnamon and other spices, mint, red pepper, herbal tooth powders, and khat [4-7]. Repeated mucosal exposure to an exogenous antigen is thought to promote local immune activation with recruitment and maturation of B lymphocytes into plasma cells within the gingival lamina propria. The resulting plasma-cell-rich infiltrate, together with associated vascular and stromal inflammatory changes, produces the erythematous enlargement and bleeding tendency characteristic of the disorder.

In the present case, directed history did not identify a consistent exposure to a suspected trigger, and formal allergy or patch testing was not performed. Therefore, the lesion was classified as idiopathic based on the available evidence. This limitation is clinically relevant because allergen identification and elimination may be curative in some cases, whereas an unidentified or persistent trigger may contribute to recurrence.

Clinical features

The characteristic gingival presentation is diffuse fiery-red erythema with edematous, often lobulated enlargement, loss of stippling, and a tendency to bleed on minimal provocation; a sharp demarcation at the mucogingival junction is a helpful, though not invariable, clue [2,15]. Symptoms, when present, include soreness aggravated by hot or spicy food or by toothpaste. The present patient exhibited the typical gingival phenotype, including generalized marginal and papillary enlargement, erythema, a multilobular surface, absent stippling, and bleeding on probing, together with the less common feature of cheilitis manifesting as diffuse upper-lip swelling [9,12]. The classically described triad of gingivitis, cheilitis, and glossitis was incomplete here, as the tongue was not reported to be involved [1]; partial expression of this syndrome is consistent with the heterogeneity of the condition.

Histopathology

Histopathology remains central to the diagnosis of plasma cell gingivitis, although no single microscopic feature is pathognomonic. The usual pattern is a dense infiltrate of mature plasma cells in the subepithelial connective tissue, often accompanied by vascular dilatation, edema, and reactive epithelial changes [2,16]. Pseudoepitheliomatous hyperplasia may be present and should not be misinterpreted as dysplasia or malignancy [2,11]. The plasma cells are expected to show mature cytomorphology and to lack features concerning for neoplasia such as marked atypia, immaturity, prominent nucleoli, or destructive tissue infiltration [2].

In this case, the biopsy showed reactive epithelial changes with pseudoepitheliomatous hyperplasia and a dense plasma-cell-rich inflammatory infiltrate. The infiltrate was prominent beneath the epithelium and extended into the underlying stroma. The plasma cells were described as cytologically mature, with eccentric nuclei, cartwheel chromatin, and abundant cytoplasm, without atypia. Immunohistochemical confirmation of polyclonality using kappa and lambda light-chain expression was not performed, which is an important limitation. Therefore, the diagnosis was based on clinicopathologic correlation, including the clinical appearance, hematological screening, benign cytomorphologic interpretation, and short-term postoperative regression.

Differential diagnosis

The breadth of the differential diagnosis is the defining challenge of this condition, and it can usefully be organized by mechanism. Among reactive and hypersensitivity processes, inflammatory plaque-associated gingival enlargement and allergic/contact gingivostomatitis are the closest clinical mimics; both may produce erythema and enlargement, but neither yields the dense plasma-cell-rich infiltrate of PCG on biopsy [2]. Plasma cell mucositis shares similar histology but involves non-gingival mucosal sites; the distinction is largely topographical, and PCG can be regarded as the gingival expression of this spectrum [2]. Hormonally influenced, puberty-associated enlargement was a legitimate clinical consideration in this adolescent, but it is plaque-dependent and lacks a plasma-cell-dominated infiltrate. Granulomatous disorders, particularly orofacial granulomatosis, were considered given the concurrent labial swelling, but they are defined histologically by non-caseating granulomas rather than by a plasma-cell infiltrate.

The most important distinctions, however, are between PCG and systemic or neoplastic disease. Leukemic gingival enlargement can mimic PCG clinically, which is why a normal total and differential leukocyte count and an unremarkable peripheral smear, as obtained here, are reassuring [2,10]. Multiple myeloma and other plasma-cell neoplasms must be excluded by demonstrating polyclonality of the infiltrate and, where clinically indicated, by appropriate systemic evaluation, because a monoclonal plasma-cell population carries an entirely different prognosis and management pathway [2]. The differential diagnoses and distinguishing features of PCG are summarized in Table 2.

**Table 2:** Differential diagnosis of plasma cell gingivitis

Condition	Key clinical clue	Distinguishing feature/investigation
Plaque-associated inflammatory enlargement	Erythema/edema related to plaque and local factors	Resolves with plaque control; non-specific chronic inflammation on biopsy
Puberty/hormonally influenced enlargement	Adolescence or pregnancy; plaque-dependent	Hormonal context; lacks dense plasma-cell infiltrate
Drug-influenced gingival enlargement	History of relevant medication	Fibrotic enlargement; drug history; no plasma-cell predominance
Allergic/contact gingivostomatitis	Temporal link to an allergen (gum, dentifrice, spice)	Improves on allergen withdrawal; history/patch testing
Plasma cell mucositis	Identical histology at non-gingival mucosal sites	Topographical distribution; same plasma-cell infiltrate
Orofacial granulomatosis/granulomatous disorders	Persistent labial or facial swelling	Non-caseating granulomas on biopsy
Leukemic gingival enlargement	Boggy enlargement, may bleed; systemic symptoms	Abnormal leukocyte counts; peripheral smear; hematology referral
Plasma-cell neoplasm (e.g., multiple myeloma)	Can mimic PCG clinically	Monoclonal infiltrate; immunohistochemistry (kappa/lambda); systemic work-up
Desquamative gingivitis	Epithelial sloughing, erythema	Vesiculobullous/lichenoid features; immunofluorescence

Diagnostic dilemma

This case was diagnostically challenging for several converging reasons. The clinical appearance was non-specific and overlapped with both a benign hormonal cause and a granulomatous process, neither of which could be confirmed or excluded on inspection alone. The associated cheilitis widened the differential toward granulomatous disease and added a second affected site whose relationship to the gingival lesion was initially unclear [12,13]. Most importantly, the clinically similar but prognostically serious possibilities of leukemia and plasma-cell neoplasia made it unsafe to proceed on a presumptive benign diagnosis [2]. The diagnostic pathway, therefore, depended on a logical exclusionary sequence: hematological screening to assess for systemic and malignant disease, followed by biopsy to establish the tissue diagnosis. The normal leukocyte indices made leukemia unlikely, and the biopsy findings supported a benign plasma-cell-rich process in the appropriate clinical context [10]. The case illustrates why PCG is properly described as a diagnosis of exclusion in which histopathology is indispensable.

Literature review

When set against the reported literature, this case is consistent with the recognized clinical and histological profile of PCG while contributing to the small body of cases featuring associated cheilitis [9,12,13]. The gingivitis-cheilitis-glossitis triad described by earlier authors and the comparable syndrome subsequently characterized frame the labial involvement seen here, although the present patient demonstrated only two components of that triad [1,8]. Previously published cases of PCG with cheilitis are few, and they vary in the duration of lip involvement, the identification or absence of a causative allergen, and the response to treatment [9,12,13]. Because glossitis was absent and the plasma-cell infiltrate was confined to the gingiva, the present case represents neither the complete gingivitis-cheilitis-glossitis syndrome nor plasma cell mucositis, but rather plasma cell gingivitis with associated cheilitis. Our patient resembles other young patients reported with concurrent gingival and labial disease but differs in the documented short-term regression of cheilitis after gingival surgery [9].

Treatment considerations

Management of PCG is not standardized, and reported approaches include identification and elimination of a suspected allergen, meticulous plaque control and scaling [17], topical, intralesional, and systemic corticosteroids [16,18], antimicrobials, and surgical excision [2,10]. In the present case, scaling and a trial of topical corticosteroids produced an inadequate response, and gingivectomy was therefore undertaken, with regression of the gingival enlargement observed at the one-month postoperative follow-up. The concurrent regression of the upper-lip swelling after gingival surgery suggests that the cheilitis may have been related, at least in part, to contact between the inflamed gingiva and the closely apposed labial mucosa [9]. Because recurrence has been reported in PCG, particularly when an underlying allergen is not identified or eliminated, structured follow-up is recommended [2,19]. Malignant transformation of PCG has not been reported, but exclusion of neoplastic mimics remains essential [2]. In this case, follow-up was limited to the one-month postoperative visit in February 2020; no post-treatment photograph was available, and no disease-directed adjuvant therapy was administered after gingivectomy. Reported treatment modalities for plasma cell gingivitis are summarized in Table 3.

**Table 3:** Treatment modalities reported for plasma cell gingivitis Note: No single modality is consistently effective; management is individualized, and recurrence is well-recognized, particularly when an underlying allergen is not removed.

Modality	Rationale	Notes
Identification and elimination of allergens	Removes the inciting hypersensitivity stimulus	First-line where a trigger is identifiable; can be curative
Non-surgical periodontal therapy (scaling/root planing)	Reduces secondary plaque-related inflammation	Adjunctive; addresses the superimposed plaque-induced component
Topical corticosteroids	Suppresses the local inflammatory/immune response	Common first-line pharmacological measure
Intralesional/systemic corticosteroids	For lesions unresponsive to topical therapy	Reserved for refractory or extensive disease
Antimicrobials	Adjunctive in selected cases	Variable, non-specific benefit
Surgical excision/gingivectomy	Removes hyperplastic/affected tissue	Used when conservative therapy fails, as in the present case
Structured follow-up	Detects recurrence	Essential, given the recognized recurrence risk

Limitations

This case has several limitations. First, immunohistochemistry for kappa and lambda light-chain expression was not performed; therefore, polyclonality could not be confirmed immunohistochemically. Second, although directed history did not identify a likely causative allergen, formal allergy testing or patch testing was not performed. Third, no radiological investigation was performed before biopsy. Fourth, follow-up documentation was limited to the one-month postoperative visit in February 2020, and a post-treatment clinical photograph was not available. Fifth, although revised photomicrographs are provided, the available histopathology images remain limited in resolution and do not include separate 4×, 10×, and 40× images. These limitations should be considered when interpreting the case. Nevertheless, the clinical presentation, hematological screening, biopsy interpretation, absence of cytological atypia, and short-term postoperative regression collectively supported a benign reactive plasma-cell process consistent with plasma cell gingivitis in the appropriate clinicopathologic context.

Clinical significance

The clinical importance of recognizing PCG is twofold. First, early and accurate diagnosis spares the patient from inappropriate treatment of a misdiagnosed condition and, more critically, prompts the exclusion of serious systemic disease that can present similarly in the gingiva [2,10]. Second, the case demonstrates the value of interdisciplinary evaluation: hematological screening, oral pathology, and, where labial or mucosal disease coexists, dermatological input together provide the framework within which a defensible benign diagnosis can be reached. For the general dental practitioner and the periodontist, the practical message is that a fiery-red, non-stippled, readily bleeding gingival enlargement that does not behave like ordinary plaque-induced disease warrants biopsy rather than continued empirical therapy [11,15].

## Conclusions

Plasma cell gingivitis with associated cheilitis is rare and may clinically overlap with granulomatous, hematologic, and plasma-cell neoplastic disease. In this adolescent patient, hematological screening and biopsy supported a benign plasma-cell-rich inflammatory process, while the available clinical, hematological, and histopathological findings argued against leukemia and a neoplastic plasma-cell disorder. The case emphasizes that persistent gingival enlargement with labial swelling warrants biopsy, careful clinicopathologic correlation, and structured follow-up, especially when the inciting allergen is not identified and long-term outcome data are unavailable.
